# Comparing automated gaze classifiers in infant looking studies: Accuracy and vulnerability to environmental factors

**DOI:** 10.3758/s13428-026-03040-x

**Published:** 2026-05-08

**Authors:** Hiromichi Hagihara, Nanako Kimura, Lorijn Zaadnoordijk, Rei Yasuda, Rhodri Cusack, Sho Tsuji

**Affiliations:** 1 Graduate School of Human Sciences, The University of Osaka, 1-2 Yamadaoka, Suita-shi, Osaka, 565-0871 Japan; 2 International Research Center for Neurointelligence (WPI-IRCN), Institutes for Advanced Study, The University of Tokyo, 7-3-1 Hongo, Bunkyo-ku, Tokyo, 113-0033 Japan; 3 Graduate School of Engineering, The University of Tokyo, 7-3-1 Hongo, Bunkyo-ku, Tokyo, 113-8656 Japan; 4 Center for Brain and Cognition, Universitat Pompeu Fabra, Barcelona, Spain; 5 Trinity College Institute of Neuroscience and School of Psychology, Trinity College Dublin, Dublin 2, College Green, Ireland; 6 Graduate School of Informatics and Engineering, The University of Electro-Communications, 1-5-1 Chofugaoka, Chofu-shi, Tokyo, 182-8585 Japan; 7 Laboratoire de Sciences Cognitives et Psycholinguistique, Ecole Normale Superieure, EHESS, CNRS, PSL University, Paris, France

**Keywords:** Infant research, Online experiments, Webcam video data, Automated gaze coding, Data quality

## Abstract

We evaluated the performance and environmental robustness of three state-of-the-art gaze classification algorithms designed for infant looking-time research: iCatcher+, OWLET, and an Amazon Rekognition-based model. Gaze classifications for each algorithm were compared to human-coded data using a novel dataset (*N* = 47), and iCatcher+ demonstrated the highest agreement (78.4–85.4%). We then investigated the effect of environmental factors commonly encountered in webcam-based home experiments with infants. We quantified six factors: distance to the camera; infants’ left–right offset; facial rotation; facial movement; facial brightness; and spatial variability in facial brightness. Suboptimal recording conditions led to performance degradation for all algorithms. Even iCatcher+, while the most accurate overall, was susceptible, particularly when facial illumination was uneven (i.e., strong brightness variability) or when the head position moved substantially. These findings provide practical insights into the selection and deployment of gaze classification tools for infant research, and can be used to optimize instructions for participants in home webcam experiments. This study contributes to improving methodological transparency and reliability in remote infant eye-tracking research.

**Supplementary Information:**

The online version contains supplementary material available at 10.3758/s13428-026-03040-x.

Developmental scientists have shown increasing interest in testing infants via remote online experiments. During online experiments, participants use their own digital device, often from their home, and respond to prompts on the screen. While some experiments require the remote presence of an experimenter via video call (synchronous testing), it is often possible to participate in online experiments via a browser (asynchronous testing). Online experiments are attractive to developmental psychologists because they allow for the recruitment of a large number of participants (Berinsky et al., 2012; Casler et al., 2013; Tran et al., 2017), potentially from more diverse backgrounds than in lab studies (Bacon et al., 2021; Rhodes et al., 2020; Scott & Schulz, 2017; Zaadnoordijk et al., 2021; Zaadnoordijk & Cusack, 2022; but see Bánki et al, 2022; Lourenco & Tasimi, 2020). Moreover, the need to specify and automate the instructions and paradigm in asynchronous experiments can also potentially improve the replicability and transparency of the protocol (Zaadnoordijk et al., 2021). Therefore, infant and child developmental scientists have increasingly been using such online experiments and have devoted efforts to developing and fine-tuning the method (e.g., Kominsky et al., 2021; Lo et al., 2021; Rhodes et al., 2020; Scott & Schulz, 2017; Tsuji et al., 2022; Zaadnoordijk et al., 2021; Zaadnoordijk & Cusack, 2022).

However, despite the potential benefits of online testing, its wider adoption is still hampered by technical challenges with regard to measurement. Most infant online studies acquire gaze data as their main outcome measure. While in the lab researchers may use eye-trackers to map the infants’ looking behavior, online testing requires manual annotation of webcam-acquired videos on a frame-by-frame basis. Although the annotated measures are often relatively simple, such as “Did the infant look at the screen or not?” or “Did the infant look to the left or the right stimulus?,” manual frame-by-frame annotation is labor-intensive, requiring considerable time and extensive training (Erel et al., 2022, 2023; Friend & Keplinger, 2008; Venker et al., 2020).

Algorithms have been developed to automatically extract gaze behavior from videos, and for adult participants they provide a decent solution (Papoutsaki et al., 2016; Zhang et al., 2019). However, infant gaze data resulting from online experiments are often more difficult to extract automatically, as infants cannot be instructed to sit still and face the screen (e.g., Dalrymple et al., 2018; Erel et al., 2022, 2023; Hessels, Andersson et al., 2015a; Hessels, Cornelissen et al., 2015b; Niehorster et al., 2018; Schlegelmilch & Wertz, 2019; Wass et al., 2014). Without an experimenter present, this may lead to various problems that will affect the quality of the video data, such as their faces not always being visible or being positioned off-center. Although several state-of-the-art automated gaze coding methods tailored for infants have been proposed (Chouinard et al., 2019; Erel et al., 2022, 2023; Werchan et al., 2022), the extent to which each algorithm is robust to such issues remains unclear. In addition to the infants’ positioning and posture, the screen size of the device used, the camera positioning, and lighting conditions might influence outcomes.

To shed light on the factors influencing gaze coding accuracy, Hagihara and colleagues (2024) tested which of the noise factors frequently found in infant video data have the largest impact on automatic gaze coding accuracy. They evaluated head tilt, off-center webcam positioning, distance to the webcam, and angle of head illumination. They invited adult participants to the lab and systematically varied these factors while controlling participants’ gaze behavior. Subsequently, they extracted the adults’ gaze behavior with automatic gaze coding algorithms and checked how their accuracy varied due to the noise factors. Their study showed that automatic gaze coding accuracy was consistently affected by a large distance to the camera and the position of the lighting source. Importantly, Bánki and colleagues (2022) showed that instructing caregivers could greatly improve such factors. They found that, after instructions, the brightness of the lighting in the videos did not significantly differ from in-lab testing (Bánki et al., 2022). Studies such as that from Hagihara and colleagues (2024) can guide adequate participant instructions and other ways to enhance video quality for automatic gaze coding. However, although Hagihara and colleagues (2024) systematically varied the noise in a way that is likely to be produced by infants, they tested these factors in an adult sample. The question remains whether the infant-like noise in adults is a sufficient proxy for infants.

The current study used an infant dataset to compare the gaze coding performance among three state-of-the-art automated classifiers tailored for infant experiments and assessed to what extent these algorithms are vulnerable to the previously identified noise factors. The first classifier, iCatcher+, was trained with infant videos and straightforwardly yielded three gaze classifications of looking “left,” “right,” and “away” (Erel et al., 2022, 2023). The second, OWLET, works optimally when combined with a calibration procedure and produces *x–y* coordinates corresponding to the monitor (Werchan et al., 2022). An Amazon Rekognition (AR)-based model was one of the first dedicated to infant online research. It required modifications and retraining for the current study to improve performance (Chouinard et al., 2019). The noise factors used were selected based on those reported in Hagihara et al. (2024), and we identified the presence of these factors in our infant video dataset detected by automatic methods.

## Methods

### Dataset

The data analyzed stem from a dataset (Tsuji et al., 2025) collected using Children Helping Science (Scott & Schulz, 2017), a platform specialized in collecting experimental infant gaze data from webcams. Infants between 1 and 12 months of age with a gestational age of over 37 weeks were recruited on the same platform. Legal guardians gave informed consent by reading the on-screen explanations and reading aloud their consent while being video-recorded. They received a $5 gift voucher for study completion. The study protocol was approved by the Life Science Research Ethics and Safety committee at the University of Tokyo (Approval No. 24–593). The dataset comprised participants for which the video recording was complete and included stimulus sounds in which human annotations had been made, all gaze classifiers functioned without errors, and data such as experimental settings and timestamps were properly aligned without technical issues (*N* = 47, 23 girls, *M*_age_ = 257.43 days, *SD* = 103.55, range = 67–392). A further 15 infants were excluded because of technical errors (*N* = 13) or extensive fussiness (*N* = 2).

**Fig. 1:**
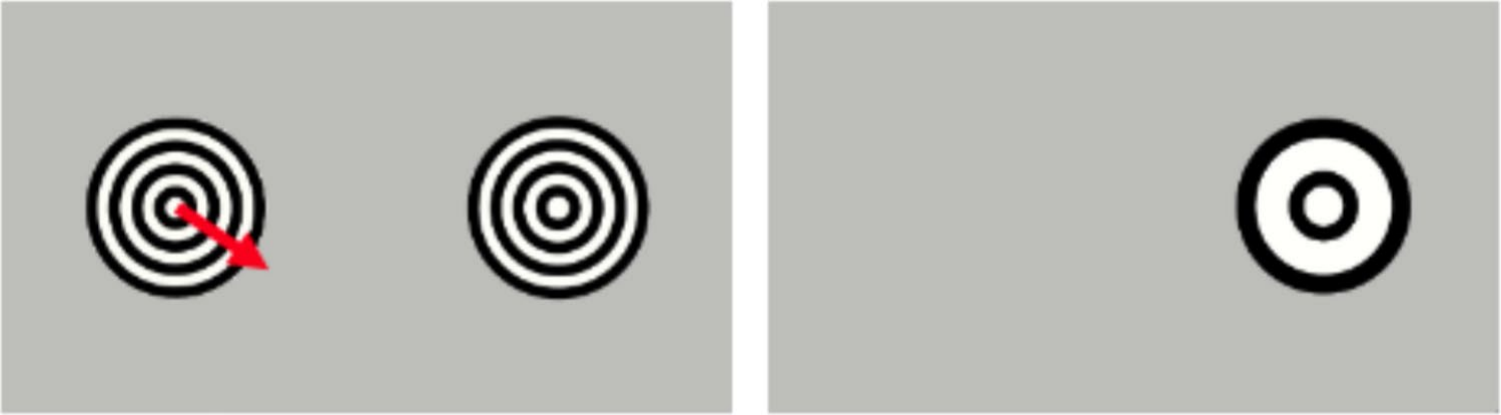
Example trial displays (black-and-white visual stimuli). *Notes*. Left: Pairwise presented high-complexity circles (red arrow symbolizes dynamic stimulus). Right: Low-complexity circle presented on one side of the screen

Infants participated in a visual preference task, in which they were exposed to black-and-white visual stimuli (see Fig. 1). Following Teller (1979), in each trial, either one stimulus was presented to the left or right of the screen (eight trials), or two stimuli were presented, one on each side of the screen (four trials). Each of the resulting 12 trials lasted for 20 s and was preceded by a 5-s central, colorful attention-getter stimulus accompanied by a chime-like sound. Within the two presentation types, we varied stimulus movement (static, dynamic) and complexity (low, high). The trial order was randomized. Between subjects, we varied stimulus shape (squares, circles) and whether there was background music present during the experiment.

Although the experimental software provided information on trial onset and other timing parameters, they did not account for delays caused by internet transmission. Therefore, to correct the onset time of each trial, we automatically detected the onset and offset of an attention-getter that preceded each trial, through its sound as recorded through the webcam. Human annotators checked and corrected this automatic detection. Infant gaze directions during each trial were coded by trained human annotators into five categories: Center, Left, Right, Away, and Unsure. Frames labeled as unsure were excluded from subsequent analyses, as these frames were considered difficult to judge even for human annotators (7.5% of all frames). In addition, frames labeled as Center were removed when calculating agreement scores, because the gaze classifiers evaluated in this study do not output a Center label. Center frames accounted for only 6.1% of all frames, so their exclusion is unlikely to affect the results. Note that these exclusions were applied only to the dependent-variable side (i.e., the computation of accuracy metrics); for the quantification of noise factors described below, we used all frames from which relevant information could be extracted. To assess the reliability of human annotations, we calculated the agreement between two trained annotators for a subset of data consisting of four participants (8.5% of the dataset, 47 trials). On average, the agreement rate was very high (*M* = 95.9%, *SD* = 5.4), ensuring that human annotations could be regarded as ground truth.

### Automated gaze classifiers

In this study, we used three state-of-the-art gaze classifiers tailored to infant looking-time experiments. Infant gaze classification for evaluation of experimental results commonly requires classification into two or three categories. In the case of two categories, the classification is between cases where the infant’s gaze is “on” or “off” screen, which is often used as a marker of general interest in the content of the screen display. In the case of three categories, the “on” category is further divided into “left” and “right” as an indicator of a preference for the content shown on the respective side of the screen. Unless otherwise specified, we used the default hyperparameter settings for each classifier.

**iCatcher+**. On the video datasets, we ran iCatcher+ v0.2.0 (Erel et al., 2023; https://github.com/icatcherplus/icatcher_plus/releases/tag/0.2.0), rooted in computer vision methods and developed especially for detecting gaze from infant online experiments. iCatcher+ was developed based on iCatcher (Erel et al., 2022), an automated gaze classifier specifically designed for research with infants and young children. iCatcher+ was reported to achieve accurate and robust gaze classification on new datasets (> 80%, Erel et al., 2023; Luchkina et al., 2025) by being trained on substantially varied datasets in terms of experimental settings (online, in-lab, and outside of the lab), research topics (intuitive physics, language comprehension), infant age, and ethnicity. iCatcher+ consists of three components: the face detector, face classifier, and gaze classifier. The face detector extracts areas that possibly include faces using OpenCV (Bradski, 2000), which was not specifically tuned toward infant face detection. Candidate areas are then fed into the face classifier. This determines whether the area is an infant’s or adult’s face and selects which of the candidate areas is most likely to belong to the participant. The gaze classifier then predicts gaze direction (i.e., Left, Right, and Away). It produces this result for the temporal middle frame within the moving window of five consecutive frames. If no face is found, the model returns a label of Invalid.

**OWLET.** This online webcam-linked eye-tracker (Werchan et al., 2022) is an open-source tool for automatically estimating infants’ gaze coordinates on a monitor based on smartphone and webcam recordings. High correlation coefficients of over .95 were reported between OWLET–human annotations in terms of overall looking time, maximum looking duration, and the number of gaze shifts. OWLET consists of three components: infants’ face/eye/pupil detector, gaze direction estimator, and estimator of point-to-gaze on the screen. The first component extracts infants’ face/gaze/pupil frame-by-frame using OpenCV (Bradski, 2000) and the Dlib Machine Learning Toolkit (King, 2009). If more than one face is extracted, the lower face is processed thereafter. The isolated pupils are fed into the gaze direction estimator, which calculates gaze direction while taking infants’ eye and head position into account. Finally, the gaze direction is mapped to precise screen (*x*, *y*) coordinates using a simple polynomial transfer function. Note that OWLET always outputs gaze coordinates based on a fixed 960 × 540 resolution, which may not match the actual screen resolution. A six-frame moving average filter is applied in this phase for smoothing, and the model produces the gaze coordinates at a temporal resolution of 30 Hz. OWLET is designed to perform optimally with the use of a four-point calibration before experiments. Accordingly, OWLET’s performance in the present study should be interpreted in light of the absence of calibration in this dataset.

In this study, we modified the source code of OWLET (downloaded March 4, 2023; https://github.com/denisemw/OWLET/tree/HEAD@{2023-03-04}) to allow processing of videos below 30 Hz because some videos had lower frame rates. Since OWLET only labels gaze as Looking or Away, we added a step to classify Looking frames into Left or Right based on the *x*-coordinate (e.g., Left if the *x* value was above 480, the midpoint).

**Amazon Rekognition (AR)-based model**. An AR-based gaze classifier (Chouinard et al., 2019; https://github.com/rhodricusack/aws_video) was trained by a video dataset collected via Children Helping Science (Scott & Schulz, 2017). The research topic of the dataset was infant language acquisition (Scott et al., 2017). Although the model’s performance in terms of looking left versus right was above chance, the developers noted that its accuracy was low ($\kappa$< 0.3 for the machine–human agreement generally), and it required substantial improvement. Using cloud-based face recognition, Amazon Rekognition (AR) (Amazon, 2022), the model first extracts features from videos such as a bounding box of the face, its estimated age, the head orientation (pitch, yaw, and roll), the position of each eye and pupil, whether the eyes are open or closed with a confidence value, and the face’s brightness and sharpness. Next, the model detects infants’ faces based on the features extracted by filtering for faces that are estimated as younger than 10 years of age. Finally, it predicts the gaze direction on a frame-by-frame basis. Note that the original version of this model had only two classes of Left or Right, but not Away, to simplify the model.

For the present study, we modified and retrained the model. We changed the output measures to three classes (i.e., Left, Right, or Away) and adopted a random forest instead of a quadratic discriminant analysis after comparing the performance of the two candidate algorithms. For the training data, we included an adult dataset that mimicked an infant experiment and systematically included noise factors (Hagihara et al., 2024) to improve the model’s robustness. Although not perfect, the model's performance improved, achieving a classification accuracy of 40.4%, which was above chance level.

### Noise factor quantification

Based on Hagihara et al. (2024), we quantified from webcam videos noise factors that could degrade the data quality: distance to the webcam, left–right offset, face rotation, face movement, face brightness, and spatial variability in face brightness. These quantifications were made using OpenFace 2.2.0 (Baltrušaitis et al. 2018), which can estimate two-/three-dimensional (2D/3D) facial landmarks and 3D head poses. We first applied OpenFace to every video and then calculated, per participant and trial, the mean distance to the webcam using the value of pose_Tz (in mm), offset using the value of pose_Tx (in mm), and face rotation using the value of pose_Rz (roll in radian). For the left–right offset and face rotation factors, the absolute values were used for the primary analysis to capture the extent of deviation from the ideal face position, regardless of direction. Larger values indicate greater displacement from the center or from upright face positions. We also measured the face movement per participant and trial by calculating the total length of face position trajectories using the values of pose_Tx and pose_Ty (in mm) within each trial. Note that if more than one face was detected for this analysis, the face in the lower position was used.

To quantify lighting conditions, we extracted two complementary brightness-related measures: overall face brightness and spatial variability in face brightness. Facial regions were first defined using OpenFace. Specifically, the left facial region was defined as the polygon enclosed by landmarks [0, 3, 5, 8, 33, 27, 21, 19, 17], and the right facial region by landmarks [16, 13, 11, 8, 33, 27, 22, 24, 26] (for landmark definitions, see https://github.com/TadasBaltrusaitis/OpenFace/wiki/Output-Format). These two regions were merged to include all pixels belonging to either the left or the right region, thereby defining the full facial region. This procedure was adopted to ensure a stable definition of the facial area. Within the resulting facial region, pixel values were converted to the CIE L*a*b color space using OpenCV v4.6.0 (Bradski, 2000) with default settings such as D65 white point (see https://docs.opencv.org/4.6.0/de/d25/imgproc_color_conversions.html), and the lightness component (L, ranging from 0 to 255) was extracted. Higher L values indicate greater brightness. Face brightness was quantified as the mean L value across the entire facial region. Spatial variability in face brightness was quantified as the standard deviation of L values within the facial region, capturing uneven illumination across the face, such as partial shading.

### Data analysis

This study evaluated model performance by calculating the agreement between human annotations and model estimates for two commonly used outcome measures in infant looking-time research: two categories (i.e., Looking or Away) and three categories (i.e., Left, Right, or Away). Agreement measures were calculated on a frame-by-frame basis, comparing labels from different sources for corresponding frames. Of the 564 trials (47 infants × 12 trials), seven trials (1.2%, from four infants) in which human annotations were entirely labeled as Unsure or Center were excluded, resulting in 557 trials (*N* = 47) for analysis. In addition, because face detection failed for one trial during the extraction of noise factors, that trial was excluded from analyses involving noise indices, resulting in 556 usable trials (*N* = 47) for those analyses.

To compare the overall performance among the three classifiers, we performed generalized linear mixed modeling (binomial regression). The dependent variable was the mean proportion of matched frames. We included the model term (i.e., iCatcher, OWLET, and AR-based model) as a fixed effect, and the trial term and the participant term as random intercepts. We also performed other generalized linear mixed models to assess to what extent each model was vulnerable to quantified noise factors. Each noise factor was standardized and then included as a fixed effect along with the model term and a two-way interaction term between the model and each noise factor. The trial and participant terms were included as random intercepts. For pairwise comparisons, we adjusted *p*-values using the Bonferroni method. The regression analysis was performed using *lme4* version 1.1.36 (Bates et al., 2015) on R version 4.4.2 (R Core Team, 2024). For statistical tests and calculating the 95% confidence intervals, we also used the packages *car* version 3.1.3 (Fox & Weisberg, 2019), *emmeans* version 1.11.0 (Lenth, 2025), *ggeffects* version 2.2.1 (Lüdecke, 2018), and *lmerTest* version 3.1.3 (Kuznetsova et al., 2017).

## Results

### Overall model performance

**Fig. 2:**
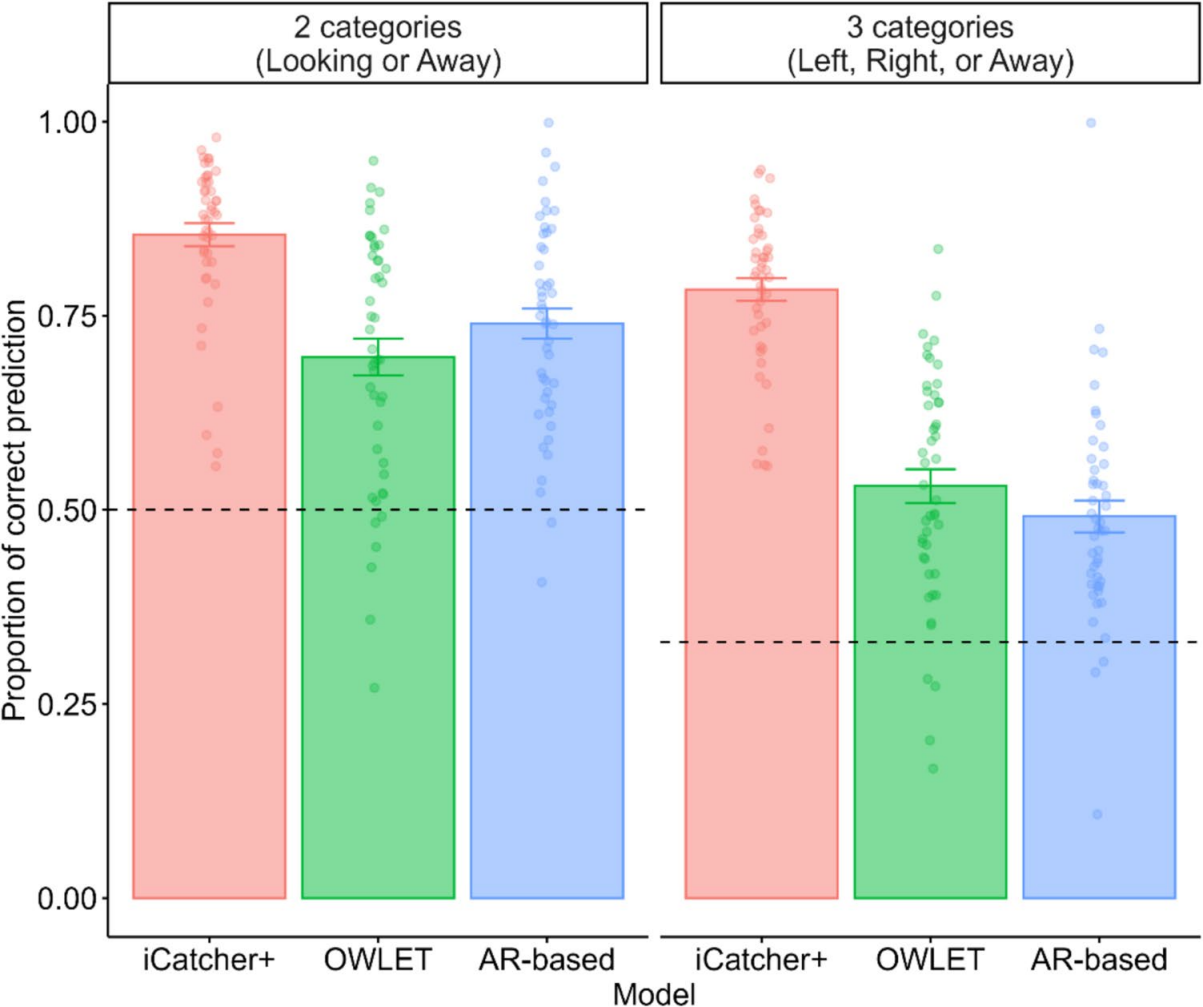
Overall human–model agreement for an infant looking-time experiment. *Notes*. Dots represent each participant. Error bars indicate standard errors. The dashed horizontal lines indicate the chance level (50% for the two-category classification and 33% for the three-category classification). For both classification types, iCatcher+ demonstrated the most superior performance, although all three models outperformed the chance level

We first assessed the overall performance among the three state-of-the-art gaze classifiers (Fig. 2). For the two-category classification, iCatcher+ demonstrated the highest overall agreement with human annotations (*M* = 85.4%, *SD* = 10.1), followed by the AR-based model (*M* = 74.0%, *SD* = 13.3) and OWLET (*M* = 69.7%, *SD* = 16.3). The regression model revealed a significant main effect of the model term, ${\chi }^{2}$(2) = 18,191, *p* < .001. According to the pairwise comparisons, iCatcher+ showed superior performance compared with the other two models (OWLET: *OR* = 2.73, *SE* = 0.02, *p* < .001; AR-based model: *OR* = 2.10, *SE* = 0.02, *p* < .001), and OWLET showed inferior performance compared with the AR-based model (*OR* = 0.77, *SE* = 0.01, *p* < .001). Given the 95% CIs from the regression model, all models outperformed the chance level (50%) (iCatcher+, 95% CI [84.8, 89.1]; OWLET, 95% CI [67.2, 75.1]; AR-based model, 95% CI [72.7, 79.6]).

For the three-category classification, iCatcher+ again demonstrated the best performance among the three models (*M* = 78.4%, *SD* = 10.0), followed by OWLET (*M* = 53.1%, *SD* = 14.9) and the AR-based model (*M* = 49.2%, *SD* = 13.9). We found a significant main effect of the model term, ${\chi }^{2}$(2) = 49,260, *p* < .001. iCatcher+ outperformed the other two models (OWLET, *OR* = 3.40, *SE* = 0.02, *p* < .001; AR-based model, *OR* = 3.81, *SE* = 0.02, *p* < .001), and OWLET performed better than the AR-based model (*OR* = 1.12, *SE* = 0.01, *p* < .001). As in the two-category classification, all three models’ performance exceeded the chance level (33%) (iCatcher+, 95% CI [77.0, 81.4]; OWLET, 95% CI [49.7, 56.3]; AR-based model, 95% CI [46.8, 53.5]).

Webcam video data collected online often vary in frames per second (fps) due to participant-specific factors and network conditions, resulting in nonuniform temporal resolution. Moreover, even in human annotations, slight timing discrepancies can occur among different annotators, particularly during moments of rapid gaze shifts. In infant looking-time studies, high temporal precision is generally not required in practice. Therefore, to avoid underestimating model performance due to such minor misalignments, we also introduced a “relaxed agreement” metric. Specifically, for each frame’s timestamp in model estimates, we defined a 100-ms target window (±50 ms) and considered it a match if the same label appeared in the human annotation within this time window. The results using this relaxed agreement metric are shown in Supplementary Fig. S1. As expected, agreement values increased slightly relative to the strict frame-based metric (e.g., for iCatcher+, from 85.4% to 86.9% in the two-category classification and from 78.4% to 80.0% in the three-category classification). The overall pattern of relative model performance remained qualitatively consistent with the strict metric.

#### Effects of noise factors on model performance

**Fig. 3:**
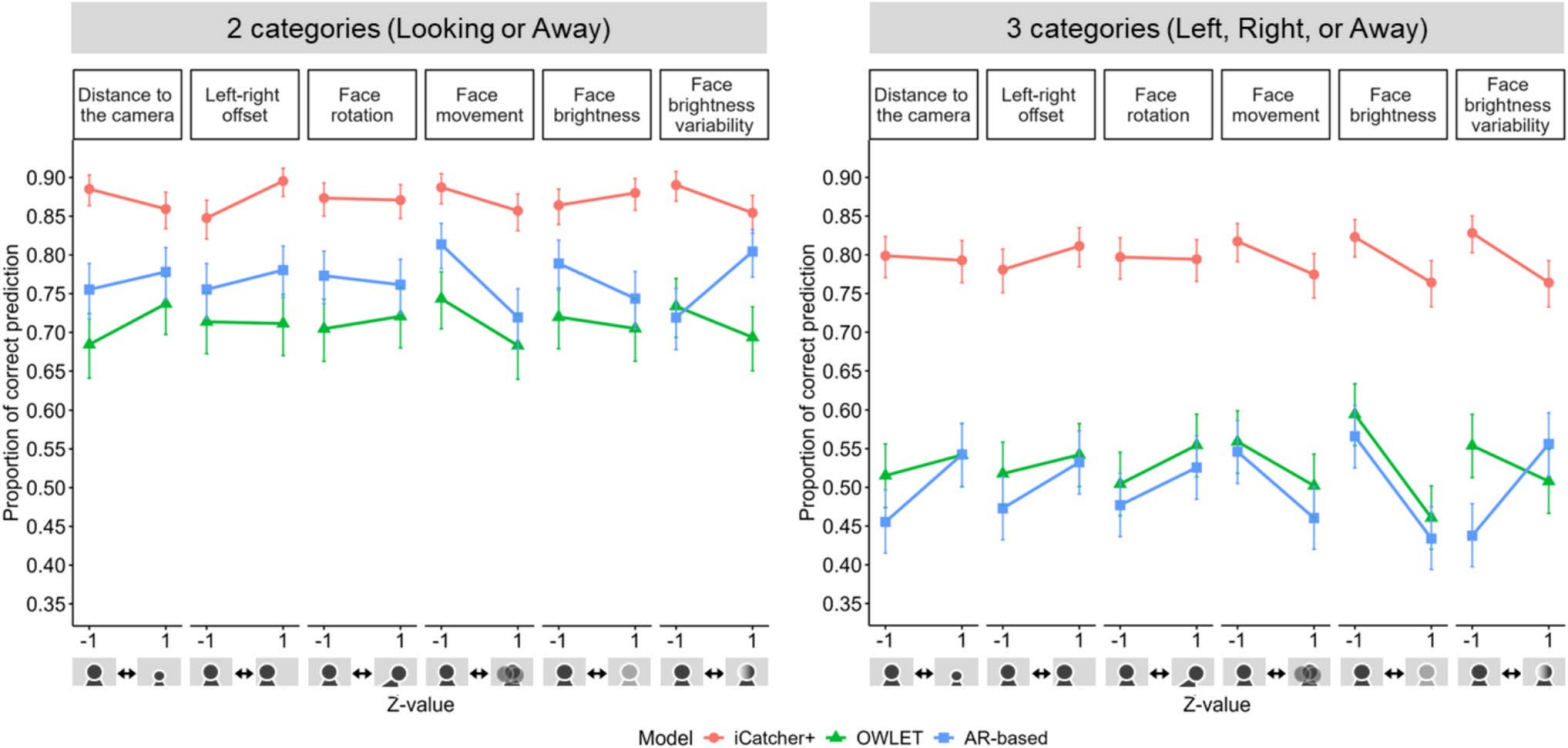
Predicted values in the models’ performance between ideal and noisy situations. *Notes*. Dots and error bars represent predicted values and 95% CIs, respectively, based on generalized linear mixed models for the two-category (left) and three-category (right) classifications. The ideal situations (–1 SD for each noise factor, except that higher values were considered ideal for face brightness) in many cases led to better performance compared with the noisy situations (+1 SD). Schematic icons beneath the *x*-axis provide intuitive illustrations of the meaning of each noise factor. Face movement consistently reduced classification accuracy across all models. For iCatcher+ and OWLET, greater face brightness variability also lowered performance, whereas for the AR-based model, uneven illumination on the face resulted in better performance. The distance to the camera often reduced accuracy for iCatcher+, but for OWLET and the AR-based model, greater distance consistently improved accuracy. The other factors showed inconsistent or sometimes opposite-to-expected effects

Next, we assessed how the candidate noise factors affected each model’s performance. For both two- and three-category classifications, our generalized linear mixed effects detected significant main effects of noise factors, except for the face rotation in the two-category classification, as well as significant interactions between noise factors and models (see Table S1 for overall results). To intuitively grasp the vulnerability of each model’s performance to each noise factor, we performed simple slope analyses, where for each quantified noise index we contrasted predicted values at –1 *SD* and +1 *SD* on the *z*-scaled noise variable, corresponding to relatively low and high levels of that factor, respectively (Fig. 3). For distance to the camera, we regarded shorter viewing distances as more ideal; for left–right offset, smaller deviations from the center; for face rotation, less head tilt; for face movement, shorter moving distance; for face brightness, greater brightness; and for face brightness variability, smaller variability. Thus, except for face brightness, lower *z*-values were considered more ideal. These definitions allowed us to interpret the direction of the effects in terms of deviation from optimal viewing conditions.

The performance of iCatcher+ demonstrated the greatest deterioration as a result of noisy brightness variability (i.e., uneven illumination on the face, such as partial shading) for both the two-category (*OR* = 1.38, *SE* = 0.04, *p* < .001) and three-category conditions (*OR* = 1.49, *SE* = 0.04, *p* < .001). Face movement also constantly worsened its performance (e.g., two-category classification, *OR* = 1.31, *SE* = 0.02, *p* < .001), and distance to the camera reduced performance in the two-category classification (*OR* = 1.26, *SE* = 0.03, *p* < .001) but only showed a marginal effect in the three-category classification (*OR* = 1.04, *SE* = 0.02, *p* = .051). The effects of the other factors were inconsistent or even opposite to expectations depending on the classification method (see Table S2 for detailed results). For OWLET, similar to iCatcher+, the noisy brightness variability and face movement consistently worsened its performance (e.g., face brightness variability for the two-category classification, *OR* = 1.22, *SE* = 0.04, *p* < .001). However, unlike iCatcher+, a longer distance to the camera led to better performance: A distance of 86 cm (+1 *SD*) was better for the model performance compared with 54 cm (–1 *SD*) for the two-category classification (*OR* = 0.77, *SE* = 0.02, *p* < .001). Face movement negatively influenced the AR-based model’s performance in particular (e.g., two-category classification, *OR* = 1.70, *SE* = 0.02, *p* < .001), and the overall tendency was roughly similar to OWLET. One salient difference was that the uneven illumination on the face resulted in better performance for both the two-category classification (*OR* = 0.62, *SE* = 0.02, *p* < .001) and the three-category classification (*OR* = 0.62, *SE* = 0.02, *p* < .001).

We initially expected overall brighter faces to be optimal. However, particularly in the three-category classification, all models showed decreased performance as the face became brighter (e.g., iCatcher+, *OR* = 1.44, *SE* = 0.03, *p* < .001). This pattern may suggest that when the face becomes overly bright, such as due to overexposure, the available facial information is reduced, and as a result, the model has difficulty extracting the visual cues needed to determine whether the infant is looking left or right.

Another aspect that may be counterintuitive was that for some noise factors, such as left–right offset, the deviation from the ideal position yielded better performance (e.g., the effect of the left–right offset on iCatcher+ for the three-category classification, *OR* = 0.83, *SE* = 0.01, *p* < .001). This could be explained by the fact that iCatcher+ sometimes showed asymmetric patterns in prediction accuracy: in some cases, Left was predicted more correctly than Right, and vice versa (Hagihara et al., 2024). To further investigate the potential asymmetric influence of the noise factors, we moved beyond using absolute values and instead took leftward and rightward deviations into account. This allowed us to examine whether deviations in different directions have differential effects on performance.

In this exploratory analysis, we focused on the three noise factors: left–right offset and face rotation. To interpret the results easily and intuitively, each factor was discretized into four groups so that the group sizes were balanced (see Table S3). Specifically, the left–right offset was divided using a threshold of ±30 mm from the center, and 5 degrees for the face rotation. We constructed new generalized linear mixed models using these discretized factors as fixed effects, while keeping all other model specifications consistent with the previous analysis (see Table S4 for the overall results of the model fittings).

**Fig. 4:**
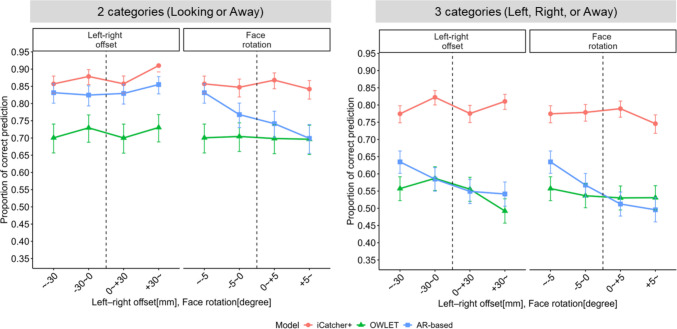
Predicted values in the models’ performance across noise factors reflecting left–right directional deviations. *Notes*. The two-category classification (left) and the three-category classification (right). Greater values indicate stronger rightward deviation (left–right offset) or rightward rotation (face rotation), from the infants’ perspectives. The dashed vertical lines represent ideal (i.e., left–right symmetric) situations

If a model performed equally well regardless of left–right deviation, the proportion of correct predictions would form a symmetrical inverted-U shape centered around the ideal value for each factor. However, this was sometimes not the case (Fig. 4). For example, in iCatcher+, the prediction accuracy for judging whether the infant was looking at the screen or not was higher when the infant was positioned to the right (> +30 mm, see Table S5 for the results of simple slope analyses). In contrast, for determining which side of the screen the infant was looking at, performance improved when the infant’s face was positioned slightly leftward (Fig. 4). In OWLET, better performance was generally observed when the infant’s face was slightly to the left of the camera. The leftward advantage was even more pronounced in the AR-based model, especially for face rotation, for which stronger leftward rotation led to relatively better performance. These directional asymmetries may help explain the seemingly counterintuitive result from our earlier analysis using absolute values, in which greater noise sometimes led to improved model performance.

## Discussion

In this study, we evaluated the performance and environmental robustness of three state-of-the-art gaze classification algorithms specifically designed for infant looking-time research: iCatcher+, OWLET, and an AR-based model. First, using a novel dataset of a webcam-based home experiment with infants, we compared the accuracy of each algorithm with human annotations. We tested performance both on a two-category classification (looking to screen vs. looking away) and on a three-category classification (looking left, looking right, looking away). While all models performed above chance, iCatcher+ outperformed OWLET and the AR-based model, noting that OWLET’s performance should be interpreted in light of the absence of calibration in the present dataset. In both cases, iCatcher+ achieved the highest agreement with human coders (an overlap of 85.4% in the two-category classification and 78.4% in the three-category classification) and, therefore, had the most reliable classification accuracy. Our results suggest that developmental scientists wishing to use online classification would be best served using iCatcher+, as has recently been reported (Luchkina et al., 2025). However, trained human annotators still show substantially higher performance than automated classification algorithms, and so careful consideration should be given on the relative costs of testing versus annotation.

Second, we investigated how environmental factors that are commonly encountered in webcam-based remote studies affected automatic gaze estimation performance. We studied how the algorithms’ performance changed as a function of distance to the camera, left–right offset in the video, head rotation, facial movements, facial brightness, and spatial variability in facial brightness. The noise factors that reduced performance included those shared across algorithms as well as those specific to each algorithm. Face movement consistently lowered classification accuracy for all three models. Uneven facial illumination (i.e., strong brightness variability) impaired performance for both iCatcher+ and OWLET, whereas its effects on the AR-based model resulted in better performance. Overly bright facial illumination in particular reduced accuracy in OWLET and the AR-based model. These findings are valuable in two ways. First, they can guide instructions given to participants. Future platforms might even be able to include the automated assessment of the noise factors into the online testing, providing real-time requests to the participant to adjust their recording conditions.^1^ Second, the factors can guide the optimization of future classification tools—either by modifying the algorithms or, more straightforwardly, by extending their training datasets to include specific manipulation of the most disruptive factors.

These results are largely in line with the findings from Hagihara et al (2024), who systematically tested the performance and vulnerability to environmental noise factors of iCatcher+ and OWLET in adults. In our results, we occasionally observed asymmetric patterns in model performance, particularly with respect to left–right directional accuracy. At least three hypotheses may account for these asymmetries. First, they may arise from biases in the training data or model overfitting, for example, if the datasets used for training contained systematic lateral biases (e.g., caregivers holding infants predominantly on one side). Second, such asymmetries may partly reflect perceptual asymmetries in humans. Lateralization in face processing and left-visual-field advantages have been documented (e.g., Harrison & Strother, 2019), and human-labeled training data may embed such perceptual biases into the models. Third, some asymmetries may simply reflect statistical noise rather than meaningful directional effects.

Relatedly, for the AR-based model, greater brightness variability unexpectedly improved classification accuracy, possibly because the model leveraged shading cues to infer whether the infant was looking toward the monitor. Since the additional training data introduced during model refinement (Hagihara et al., 2024) included many examples with strong lateral illumination, this pattern may reflect characteristics of the enhanced training set. Regardless of the explanations discussed here, there is no fundamental method to correct such asymmetry-induced distortions. Thus, when using automated gaze coding tools, it may be advisable to conduct complementary analyses with human annotations for a subset of the data to confirm that these biases do not substantially compromise the annotation estimates. Future work should more systematically evaluate which of the above explanations is most likely and examine whether similar asymmetries are replicated in newly collected datasets.

The effects of the distance to the camera varied depending on the classifier used and the number of classification categories. For iCatcher+, shorter distances often improved accuracy, whereas for OWLET and the AR-based model, accuracy tended to improve when infants were farther from the camera. These differences may stem from how each model processes facial information. For instance, iCatcher+ uses full-face image information and face-region size extracted via OpenCV (Bradski, 2000). OWLET relies on OpenCV and the Dlib Machine Learning Toolkit (King, 2009) to extract face, gaze, and pupil features. It is also possible that the factor affecting accuracy is not distance itself but the pixel density of the facial region. Indeed, in our dataset, greater distance was associated with a smaller proportion of the image area occupied by the face (*r* = –.77, *p* < .001; see Fig. S2). Thus, when high-resolution cameras are available, adjusting the distance may not be essential; post hoc spatial cropping could be used for stable gaze estimation even when the face is far from the camera. For online data collection, however, practical guidance regarding camera distance may often be encouraged.

Beyond classifier-specific sensitivities to noise, another source of ambiguity arises from the video recordings themselves. Although we analyzed frames that were considered interpretable by human coders by excluding the Unsure category, it is still possible that, particularly under challenging conditions, disagreement between human annotations and automated estimates reflects indeterminacy in the visual input rather than misclassification by the model per se. Indeed, our exploratory analysis showed that human–human agreement also tended to decrease under similar adverse conditions (see Supplementary Fig. S3). Such an inherent limitation of gaze estimation in noisy, naturalistic visual data may lead to an underestimation of model performance. Practically speaking, however, the implications remain the same: researchers are encouraged to either provide clearer instructions to participants to improve recording quality or further develop models that are robust to noisy visual input.

To evaluate the impact of temporal alignment on agreement estimates, we computed a relaxed 100-ms windowed metric in addition to a conventional, strict frame-by-frame metric. Across all classifiers, the two measures produced highly similar accuracy values, differing by only a few percentage points, and the effects of environmental noise factors were qualitatively compatible across both metrics. These convergent patterns suggest that our conclusions do not depend on the choice of agreement metric and that no model disproportionately benefited from the relaxed windowing procedure. At the same time, the absolute magnitude of agreement could, in principle, vary with the size of the temporal window, particularly because different classifiers apply different forms of internal temporal smoothing. For instance, iCatcher+ takes five consecutive frames as input and estimates the gaze label for the middle frame (Erel et al., 2023). Selecting a time window that matches this scale could, in theory, give iCatcher+ an unintended advantage, although this was not the case here, as results remained robust even under strict frame-based scoring. To address variability in sampling rates across videos, future work may also consider resampling videos before annotation so that their temporal resolutions are matched.

Gaze coding algorithms offer a significant reduction in resources required to annotate gaze data from infants. Our results, together with those of Hagihara et al. (2024), show that they are a promising tool for developmental science. However, it is essential to be aware of their limits. Their accuracy depends on the chosen algorithm, the experimental design, and environmental factors. Furthermore, research questions and designs have varying demands on the precision of gaze coding. Researchers must take all these factors into consideration when deciding whether to use gaze coding algorithms instead of manual coding. As a second-best approach, one possibility is to use an automatic annotation model as a screening tool and then have human annotators verify (and correct if necessary) the results. This procedure can improve coding efficiency compared to relying solely on humans. In fact, human–machine interactions have been suggested for data annotations in other domains, such as language or image evaluation tasks (e.g., Kim et al., 2024; Subramanya et al., 2025).

In sum, this study highlights both the promise and the limitations of current automated gaze classification tools for infant looking-time research. The study contributes to improving methodological transparency and reliability in remote infant eye-tracking research by systematically assessing gaze coding performance among automated classifiers and their vulnerability to environmental noise factors. Researchers can leverage insights obtained to make more informed decisions about how to integrate these automated tools into developmental studies.

## Supplementary Information

Below is the link to the electronic supplementary material.

Supplementary file 1 (PDF 522 KB)

## Data Availability

The video data used in the present study are safely stored at the IRCN Babylab, University of Tokyo. Caregivers could consent to share their data publicly, with other research teams, or not at all. Accordingly, upon request, a subset of the data can be shared with other research teams. The anonymized gaze coding data, representing partial data from participants who consented to scientific use (*N* = 24), can be found at 10.17605/OSF.IO/HPRC2.

## Footnotes

1 For instance, RealEye, a webcam-based eye-tracking system, provides a message like “go back to the original position” whenthe participant’s head movement is excessive (https://www.realeye.io/features/online-webcam-eyetracking).

## Code availability

The codes for data preprocessing and analysis are available at https://github.com/hagi-hara/infant-gaze-classifier-eval

## Declarations

### Open practices statement

The anonymized gaze coding data, representing partial data from participants who consented to scientific use (*N* = 24), can be found at 10.17605/OSF.IO/HPRC2. The codes for data preprocessing and analysis are available at https://github.com/hagi-hara/infant-gaze-classifier-eval. None of the experiments were preregistered.
