# Supplementary material for: Comparing automated gaze classifiers in infant looking studies: Accuracy and vulnerability to environmental factors
Source: Behav Res Methods. 2026 May 8;58(6):160. doi: 10.3758/s13428-026-03040-x (PMC13156148; doi:10.3758/s13428-026-03040-x)
Supplement: Supplementary file 1 — (PDF 522 KB) [file 13428_2026_3040_MOESM1_ESM.pdf]

Supplementary Materials

Comparing automated gaze classifiers in infant looking studies:

Accuracy and vulnerability to environmental factors

Figure S1

Overall human–model agreement for an infant looking-time experiment (relaxed agreement)

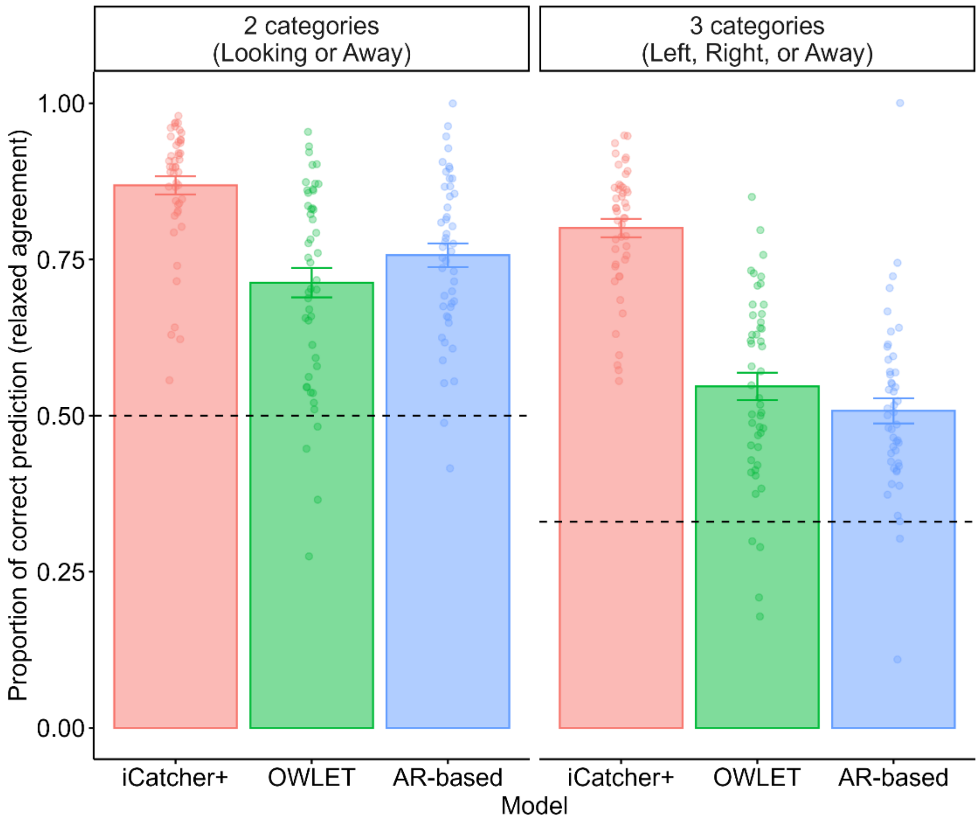

Notes. Agreement between model estimates and human annotations using the relaxed agreement metric. The relaxed agreement metric was computed by defining a 100-ms target window around each model-predicted frame and counting a match when the same label appeared in the human annotation within that window. For the two-category classification, iCatcher+ showed the highest agreement (*M*=86.9%, *SD*=9.9), followed by the AR-based model (*M*=75.7%, *SD*=12.9) and OWLET (*M*=71.3%, *SD*=16.1). For the three-category classification, iCatcher+ again performed best (*M*=80.0%, *SD*=10.1), followed by OWLET (*M*=547%, *SD*=15.1) and the AR-based model (*M*=50.8%, *SD*=13.8). Overall, the relative performance patterns were qualitatively consistent with those observed using the strict frame-based agreement metric reported in the main text. The plotting specifications and legend follow those of Figure 2 in the main text.

Figure S2

Relationship between distance to the camera and the proportion of the facial image area

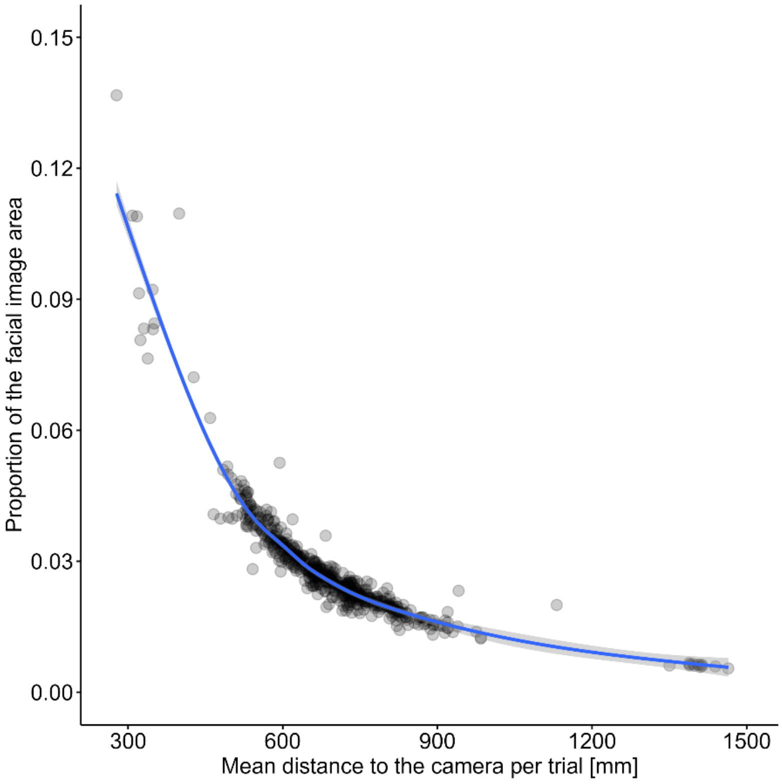

Notes. Each dot represents a trial. Of the 47 participants, most recordings were captured at 1,280 × 720 px (16:9; N=44), and the remaining three at 640 × 480 px (4:3). A smoothed regression curve estimated via GAM is overlaid, with the 95% CI shown as a gray ribbon. A strong negative correlation was observed between the two measures (r=–.77, p<.001). For the definition of the facial region, see the Noise factor quantification section in the main text.

Figure S3

Predicted human–human agreement in ideal vs. noisy situations.

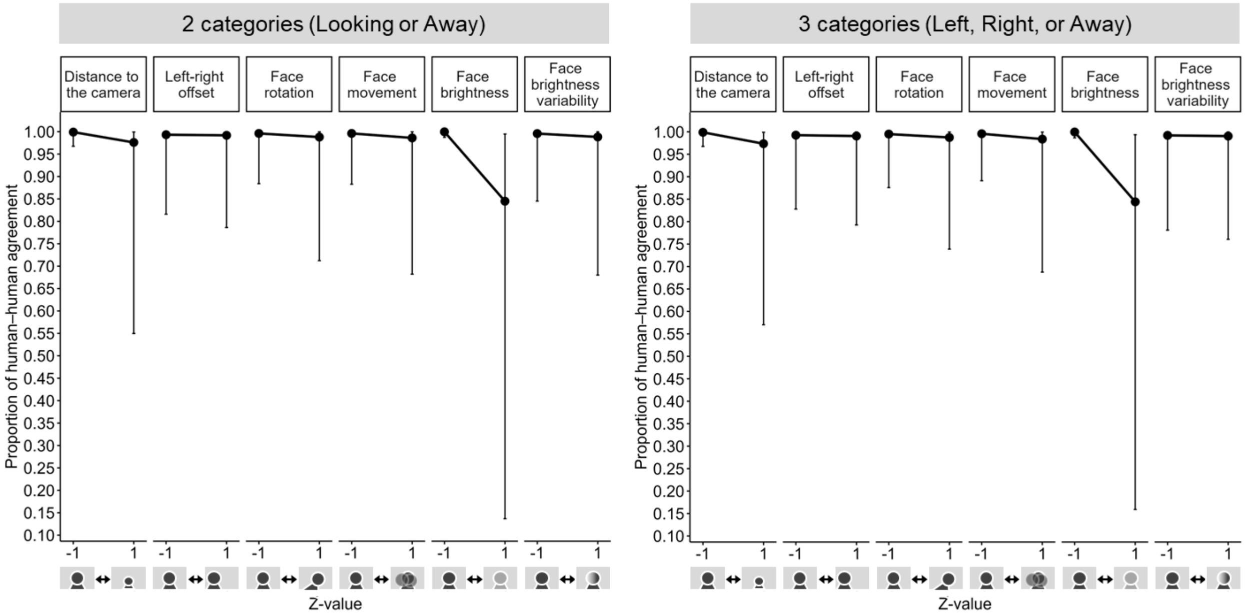

Notes. We applied the same GLMMs used in the main analysis to the subset of data used for assessing inter-annotator reliability (*N*=4 participants, 46 trials). One trial was excluded because OpenFace failed to detect the face. Overall, the patterns were similar to those observed for human–model agreement. In both the two-category and three-category classifications, distance to the camera, face rotation, and face movement reduced human–human agreement (*p*s<.001). Higher overall facial brightness also tended to decrease agreement (*p*s<.001). In contrast, left–right offset and face brightness variability did not have significant effects (*p*s>.071). The plotting specifications and legend follow those of Figure 3 in the main text.

Table S1

Overall results of the effects of noise factors on models' performance.

	Two-category classification		Three-category classification	
Fixed effect	χ^2	p-value	χ^2	p-value
Model	17616.17	< .001	47930.59	<.001
Distance to the camera	30.06	< .001	108.20	<.001
Left–right offset	191.67	< .001	456.50	<.001
Face rotation	0.01	.94	312.65	<.001
Face movement	2240.03	< .001	1498.55	<.001
Face brightness	17.55	< .001	642.17	<.001
Face brightness variability	2.41	.12	0.72	.39
Model × Distance to the camera	876.91	< .001	807.92	<.001
Model × Left–right offset	622.06	< .001	115.39	<.001
Model × Face rotation	130.38	< .001	357.26	<.001
Model × Face movement	392.12	< .001	86.52	<.001
Model × Face brightness	564.41	< .001	172.86	<.001
Model × Face brightness var.	2971.62	< .001	5050.52	<.001

Notes. Anova function (Type II Wald chi-square tests) in package *car* version 3.1.3 was used (Fox & Weisberg, 2019). All noise factors were standardized and then fit to the model. For the left–right offset and face rotation, the absolute values were used.

Table S2

Comparisons of the effects of noise factors between the ideal and noisy conditions.

	Two-category classification			Three-category classification		
	Odds ratio	*SE*	*p*-value	Odds ratio	*SE*	*p*-value
iCatcher+						
Distance to the camera	1.26	0.03	<.001	1.04	0.02	.051
Left–right offset	0.65	0.01	<.001	0.83	0.01	<.001
Face rotation	1.02	0.01	.080	1.01	0.01	.14
Face movement	1.31	0.02	<.001	1.30	0.02	<.001
Face brightness	0.87	0.02	.016	1.44	0.03	<.001
Face brightness variability	1.38	0.04	<.001	1.49	0.04	<.001
OWLET						
Distance to the camera	0.77	0.02	<.001	0.90	0.02	<.001
Left–right offset	1.01	0.01	.32	0.91	0.01	<.001
Face rotation	0.93	0.01	<.001	0.82	0.01	<.001
Face movement	1.35	0.01	<.001	1.26	0.01	<.001
Face brightness	1.08	0.03	.0017	1.72	0.04	<.001
Face brightness variability	1.22	0.04	<.001	1.20	0.03	<.001
AR-based model						
Distance to the camera	0.88	0.02	<.001	0.71	0.01	<.001
Left–right offset	0.87	0.01	<.001	0.79	0.01	<.001
Face rotation	1.07	0.01	<.001	0.82	0.01	<.001
Face movement	1.70	0.02	<.001	1.41	0.01	<.001
Face brightness	1.29	0.03	< .001	1.70	0.04	<.001
Face brightness variability	0.62	0.02	<.001	0.62	0.02	<.001

Notes. Simple slope analyses were performed between the ideal (–1*SD*, except for face brightness) and noisy (+1*SD*) values in each noise factor for each gaze classifier.

Table S3

Discretization of noise factors.

	#Datapoint	*M*	*SD*	Range
Left-right offset [mm]				
Group 1: < –30	226	–79.0	52.4	[–429.0, –30.1]
Group 2: –30 ~ 0	117	–15.7	8.3	[–30.0, –0.2]
Group 3: 0 ~ +30	95	12.9	8.1	[0.2, 29.4]
Group 4: +30 <	118	77.9	51.3	[30.4, 276.0]
Face rotation [degree]				
Group 1: < –5	94	–8.3	3.1	[–19.2, –5.1]
Group 2: –5 ~ 0	178	–2.3	1.5	[–5.0, 0.0]
Group 3: 0 ~ +5	177	2.5	1.4	[0.0, 4.9]
Group 4: +5 <	107	10.6	6.4	[5.0, 30.6]

Notes. Regarding the left–right offset, the further to the right, the greater the positive value, from the baby's perspective. Similarly, as for the face rotation, greater values indicate face tilting (roll) toward the right.

Table S4

Overall results of the effects of noise factors on models' performance (discretized indices).

	Two-category classification		Three-category classification	
Fixed effect	χ^2	p-value	χ^2	p-value
Model	17908.48	<.001	48283.73	<.001
Left–right offset	645.32	<.001	572.36	<.001
Face rotation	441.35	<.001	574.37	<.001
Model × Left–right offset	554.28	<.001	1937.95	<.001
Model × Face rotation	1480.62	<.001	1254.11	<.001

Notes. Each noise factor was discretized into four groups (see Table S3).

Table S5

Comparisons of the effects of noise factors (discretized).

	Two-category classification	Three-category classification
iCatcher+		
Left–right offset	Gr.1 ≈ Gr.3 < Gr.2 < Gr.4	Gr.1 ≈ Gr.3 < Gr.4 < Gr.2
Face rotation	Gr.4 ≈ Gr.2 < Gr.1 < Gr.3	Gr.4 < Gr.1 ≈ Gr.2 < Gr.3
OWLET		
Left–right offset	Gr.3 ≈ Gr.1 < Gr.4 ≈ Gr.2	Gr.4 < Gr.3 ≈ Gr.1 < Gr.2
Face rotation	Gr.4 ≈ Gr.3 ≈ Gr.1 ≈ Gr.2	Gr.4 ≈ Gr.3 ≈ Gr.2 < Gr.1
AR-based model		
Left–right offset	Gr.2 ≈ Gr.3 ≈ Gr.1 < Gr.4	Gr.4 ≈ Gr.3 < Gr.2 < Gr.1
Face rotation	Gr.4 < Gr.3 < Gr.2 < Gr.1	Gr.4 < Gr.3 < Gr.2 < Gr.1

Notes. Simple slope analyses were performed for all the group pairs. Higher group numbers indicate stronger rightward deviation (Left–right offset) or rightward rotation (Face rotation). Inequality signs "<" represent significant differences, while "≈" signs indicate non-significance. *P*-values were adjusted using the Bonferroni method.
